# Metabolomic and transcriptomic analyses reveal the effects of grafting on blood orange quality

**DOI:** 10.3389/fpls.2023.1169220

**Published:** 2023-06-01

**Authors:** Lei Yang, Yang Chen, Min Wang, Huifang Hou, Shuang Li, Ling Guan, Haijian Yang, Wu Wang, Lin Hong

**Affiliations:** ^1^Fruit Tree Research Institute, Chongqing Academy of Agricultural Sciences, Chongqing, China; ^2^Biotechnology Research Institute, Chongqing Academy of Agricultural Sciences, Chongqing, China

**Keywords:** blood orange, flavonoids, pigmentation, gene expression, quality improvement

## Abstract

**Introduction:**

Blood orange (*Citrus sinensis* L.) is a valuable source of nutrition because it is enriched in anthocyanins and has high organoleptic properties. Grafting is commonly used in citriculture and has crucial effects on various phenotypes of the blood orange, including its coloration, phenology, and biotic and abiotic resistance. Still, the underlying genetics and regulatory mechanisms are largely unexplored.

**Methods:**

In this study, we investigated the phenotypic, metabolomic, and transcriptomic profiles at eight developmental stages of the lido blood orange cultivar (*Citrus sinensis* L. Osbeck cv. Lido) grafted onto two rootstocks.

**Results and discussion:**

The Trifoliate orange rootstock provided the best fruit quality and flesh color for Lido blood orange. Comparative metabolomics suggested significant differences in accumulation patterns of metabolites and we identified 295 differentially accumulated metabolites. The major contributors were flavonoids, phenolic acids, lignans and coumarins, and terpenoids. Moreover, transcriptome profiling resulted in the identification of 4179 differentially expressed genes (DEGs), and 54 DEGs were associated with flavonoids and anthocyanins. Weighted gene co-expression network analysis identified major genes associated to 16 anthocyanins. Furthermore, seven transcription factors (*C2H2*, *GANT*, *MYB-related*, *AP2/ERF*, *NAC*, *bZIP*, and *MYB*) and five genes associated with anthocyanin synthesis pathway (*CHS*, *F3H*, *UFGT*, and *ANS*) were identified as key modulators of the anthocyanin content in lido blood orange. Overall, our results revealed the impact of rootstock on the global transcriptome and metabolome in relation to fruit quality in lido blood orange. The identified key genes and metabolites can be further utilized for the quality improvement of blood orange varieties.

## Introduction

Blood oranges (*Citrus sinensis*) with distinctive crimson flesh are enriched with anthocyanins (Moufida and Marzouk, 2003; Butelli et al., 2012). The flesh color in blood orange mainly depends on the accumulation of anthocyanins (Habibi et al., 2022). In combination with vitamin C, carotenoids, and fiber, anthocyanins provide a healthy addition to the blood orange dietary properties (Prior and Wu, 2006; De Pascual-Teresa et al., 2010; Paredes-López et al., 2010). Striking color, nutritional properties, and enriched anthocyanins favored blood oranges for health-promoting properties (Kelebek et al., 2008). Anthocyanin-enriched food with high antioxidant activity has distinctive properties against certain diseases such as cancer, heart disease, cholesterol accumulation, and obesity (Hou, 2003; De Pascual-Teresa et al., 2010; Paredes-López et al., 2010; Titta et al., 2010). However, the color and anthocyanin accumulation in blood orange highly depends on the cultivar, environmental attributes, soil, agronomic practices, and rootstock (Rapisarda et al., 2001; Crifò et al., 2011; Siracusa and Ruberto, 2014). Several studies focused on anthocyanin accumulation, composition, and underlying biological pathways in blood oranges have demonstrated potential for quality improvement (Li et al., 2014; Cebadera-Miranda et al., 2019). Aside from anthocyanin enrichment and flesh color, several quality parameters influence consumer choices. These factors include but are not limited to firmness, texture, sugar contents, acids contents, and shelf life (Pallottino et al., 2013; Fabroni et al., 2020).

Citriculture takes advantage of the grafting, and published literature suggests a strong influence of rootstocks on economic traits, such as fruit yield, size, quality, maturation, and postharvest performance (Rodríguez-Gamir et al., 2010; Bowman et al., 2016; Forner-Giner et al., 2020). However, the effect of rootstocks on blood oranges is not well established, with few studies. For instance, Morales et. al., used eight different rootstocks and identified significant differences in metabolite accumulation patterns, specifically phenolics in two orange cultivars (Morales et al., 2021). Another study by Modica et. al., demonstrated differential accumulation patterns of polyphenols, especially anthocyanins, in blood oranges grafted on ten different rootstocks under different environmental conditions (Modica et al., 2022). As fruit quality is highly dependent on genotype and environmental attributes, it is necessary to decipher the underlying mechanism specific to genotype under certain environmental influences.

In recent years, combining omics for integrative analysis of a phenomenon gives more robust outcomes to decipher the underlying genetic mechanisms (Subramanian et al., 2020; Nazir et al., 2021; Raza et al., 2021). A combination of metabolomics and transcriptomics has been widely adapted in horticulture, yielding potential insights for further use in breeding programs for improvement (Su et al., 2019; Tahir Ul Qamar et al., 2020; Zhang and Hao, 2020; Gong et al., 2021). For instance, Gong et. al., utilized an integrative approach combing transcriptomics and metabolomics to understand the flavor of crimson-colored watermelon resulting in the identification of five candidate genes in the glycolytic pathway favoring flavor enhancement (Gong et al., 2021). Although few studies have addressed the quality attributes of red blood oranges in response to different rootstocks, Chinese red blood cultivars have not been characterized for changes in quality parameters due to different rootstocks. Therefore, evaluating existing germplasm and understanding the regulatory mechanisms for utilization in further breeding programs is necessary.

The current study aimed at deciphering the molecular mechanism underlying differential metabolic profiles of the lido blood orange cultivar, which were grafted on four different rootstocks. Lido blood orange fruits at eight different stages were characterized for metabolic and transcriptomic profiles, and conjoint analysis of metabolome and transcriptome further narrowed down the significant variations concerning the fruit quality of lido red blood oranges.

## Materials and methods

### Plant material and sample collection

Lido blood orange variety grafted on four rootstocks, ‘Trifoliate orange’ (*P. trifoliata* L. Raf., Pt), ‘Ziyang Xiangcheng’ (*C. junos* Sieb. ex Tanaka, Cj), *C. reticulata* Blanco, and ‘Citrange’ (*Poncirus trifoliate* L. raf. × *Citrus sinensis* L. Osbeck.) were used in this study. These four rootstocks are hereinafter referred to as Z, X, H, and ZC, respectively. The experimental orchard was established at the regional test base of citrus varieties of Chongqing Academy of Agricultural Sciences, Jiangjin District, Chongqing, China (116° 34 ′ E, 36° 50 ′ N). The experimental region is located in a warm, humid monsoon climate. The region experiences an average annual temperature of 18.4°C, 1001.2 mm of precipitation, 805.5 mm of evaporation, and 206 days without a frost. The citrus orchard in the research region has purple soil type and sandy loam soil texture (6% sand, 89% silt, and 5% clay) with pH 6.43, available potassium 103.83 mg.kg^-1^, available calcium 0.61 g.kg^-1^, available magnesium 0.09 g.kg-1, available copper 2.78 mg.kg^-1^, available iron 191.80 mg.kg^-1^, available manganese 9.67 mg.kg-1, available zinc 3.47 mg.kg^-1^, and available boron 1.05 mg.kg^-1^. The fertilization rate is 1 kg of compound fertilizer plus 0.5 kg of urea plus 1.5 kg of organic fertilizer per plant; before the middle of July in the summer, apply fertilizer for fruit expansion; 1 kg of high potassium compound fertilizer per plant is applied in the ditch; and water-soluble middle and trace elements are supplemented on the leaves.

Fruits were obtained from 4 different rootstocks at eight-time points (harvesting time from October to February). The time points for fruit sampling were T1 (October 12), T2 (November 17), T3 (November 30), T4 (December 15), T5 (December 30), T6 (January 1), T7 (January 29), and T8 (February 15). The tree age was six years at the sampling time, and the planting density was 3 m × 4 m. Three replicates were used for each sample group. Nine blood orange trees with the same growth and similar fruit-bearing capacity were selected as the sample trees from 20 Lido blood oranges with different rootstocks. One sample fruit was collected from the southeast and northwest of each sample tree. The 15 fruits of each three sample trees were mixed into one biological sample of three sets. After the 15 fruits of each biological replicate were transversely cut, we used a scalpel to pick up a small amount of pulp, mix it evenly, and use a juicer to extract juice from the rest. The pulp is used for real-time quantitative analysis of the transcriptome, metabolome, and candidate genes, and the juice was used for the determination of the total solid solids (TSS), vitamin C (VC), acid concentration (A-conc.) and other basic qualities.

The appearance color, soluble solids to acidity ratio, acid concentration, total soluble solids, and vitamin C contents of each replicate fruit were determined. The total soluble solids were measured using a refractometer (Yu et al., 2022) and acid concentration (A-conc.) by acid-base titration by Norminkoda Biotechnology Co., Ltd., Wuhan, China. The Vitamin C was extracted and quantified using the plant ascorbic acid content detection kit (Norminkoda Biotechnology Co., Ltd. Wuhan, China) (Sun et al., 2023).

### Metabolomic profiles

Metabolomic profiling was performed by Wuhan Metware Biotechnology Co., Ltd (https://www.metware.cn) following the company’s standard procedures with a series of procedures, including extraction, identification, and quantification of metabolites (Cao et al., 2019; Wang et al., 2019). Cryo-preserved fruit samples (juice and pulp mixture) were weighed and extracted with 1.0 ml of 70% methanol. The methanol extracts were subjected to liquid chromatography mass-spectrometry/M.S. analysis (LC-MS/MS, UPLC, Shim-pack UFLC SHIMADZU CBM30A system; MS, Applied Biosystems 6500 QTRAP). Metware’s metabolite database and public metabolite database were used to identify the metabolites, and quantification was done accordingly. To further access, the differential accumulation patterns of metabolites between different samples were determined using orthogonal partial least squares discriminant analysis. Metabolites with |Log_2_ Foldchange| ≥ 1 and VIP (variable importance in project) ≥ 1 were defined as DAMs.

### Transcriptomic profiles

Total RNA was extracted from the samples (with three biological replicates) with the RNA Extraction kit (TIANGEN, Beijing, China). The RNA quality and concentration were assessed with agarose gel electrophoresis and NanoDrop2000 spectrophotometer. Quality testing, library construction, and sequencing for each sample were done at Metware Biotechnology Co., Ltd (https://www.metware.cn), following the company’s standard procedures. Low-quality data were removed for downstream analysis, and high-quality clean reads were used for transcriptome quantification. The clean reads were localized using Hisat2 to obtain unigenes (Sirén et al., 2014). Reads per kilobase mapping (FPKM) for all genes were used to determine gene expression values, and further screening for differentially expressed genes (DEGs) was performed. The differentially expressed genes (DEGs) were identified by R package DESeq2 for subsequent analysis. The genes featuring FDR < 0.05 and |Log_2_ Fold change|≥ 1 were considered DEGs. The identified DEGs were further enriched by KEGG analysis. Gene Ontology (GO) annotation and Kyoto Encyclopedia of Genes and Genomes (KEGG) pathway enrichment analysis were applied using Tbtools software (Chen et al., 2020). Heat maps were generated using the OmicStudio tools at https://www.omicstudio.cn/tool.

### RT-qPCR based verification of expression of candidate genes

Total RNA was reverse-transcribed in a 20 μL reaction mixture using the HiScript^®^ II Q RT SuperMix for qPCR (+gDNA wiper) kit (Vazyme). The 20 μL reactions were performed with 10 μL of ChamQTM SYBR^®^ qPCR Master Mix (High ROX Premixed), 1.0 μL 10 mM forward and reverse primers, 7.0 μL of ddH_2_O and 2.0 μL 5 times diluted cDNA template. The Citrus Actin and EF1 α were used as internal reference genes. The ABI Prism 7500 Fast system was used to perform RT-qPCR. The primers used for RT-qPCR are listed in **Supplementary Table 8**. The relative expression of genes was calculated by the 2^-ΔΔCT^ method (Schmittgen and Livak, 2008).

### Identification of hub genes using WGCNA analyses

Weighted Gene Correlation Network Analysis (WGCNA) (Fuller et al., 2007) was used to identify hub genes. WGCNA analysis was performed with WGCNA-shiny in TBtools (Toolbox for Biologists, v1.082). Cluster analysis was performed on the samples according to the expression levels of DEGs. The correlation between the characteristic gene of the module and the trait was calculated, including the correlation between the gene and the characteristic expression in the module (module membership, MM) and the correlation between each gene and the target trait (gene significance, GS). We used the passing threshold GS.abs >0.5 and MM.abs >0.8 to identify the hub gene of each module. Transcription factor (TF) annotations were searched for the hub genes using PlantTFDB (Jin et al., 2016). Cytoscape (Cytoscape Consortium, USA) software was used to visualize the gene interaction network.

## Results

### Effect of grafting on physiological and color parameters in lido blood orange

Physiological parameters including total soluble solids (TSS), vitamin C (VC), acid concentration (A-conc.), soluble solids to acidity ratio (SS), and color parameters L (brightness), a (red/green value), and b (Blue/yellow value) from fruit samples were estimated in lido blood orange grafted on four rootstocks Z (Trifoliate orange), ZC (Citrange), H (*C. reticulata* Blanco), and X (Ziyang Xiangcheng). The data was collected at 8 different time points from October 2021 to February 2022 (T1 to T8) and subjected to analysis of variance. All physiological parameters were significantly different (P < 0.005) between the four rootstocks, implying that the rootstocks altered the nutritional composition of the fruits (**Tables S1**, **S2** and **Figure S1**). Similarly, we observed a significant effect of the sampling time on the physiological parameters of the fruits. Concerning the flesh color parameters, only L values differed significantly (P < 0.05) between the rootstocks (**Tables S1**, **S2** and **Figure S1**). However, the sampling time significantly affected all color parameters, indicating that the fruit color changed considerably over the sampling period.

Collectively, fruits samples from Z and X were the most contrasted in terms of color and physiological parameters (**Figure S3**), therefore, they were selected for downstream analyses.

### Differential metabolic profile of lido blood orange grafted to two rootstocks (Z and X)

**Figure 1:**
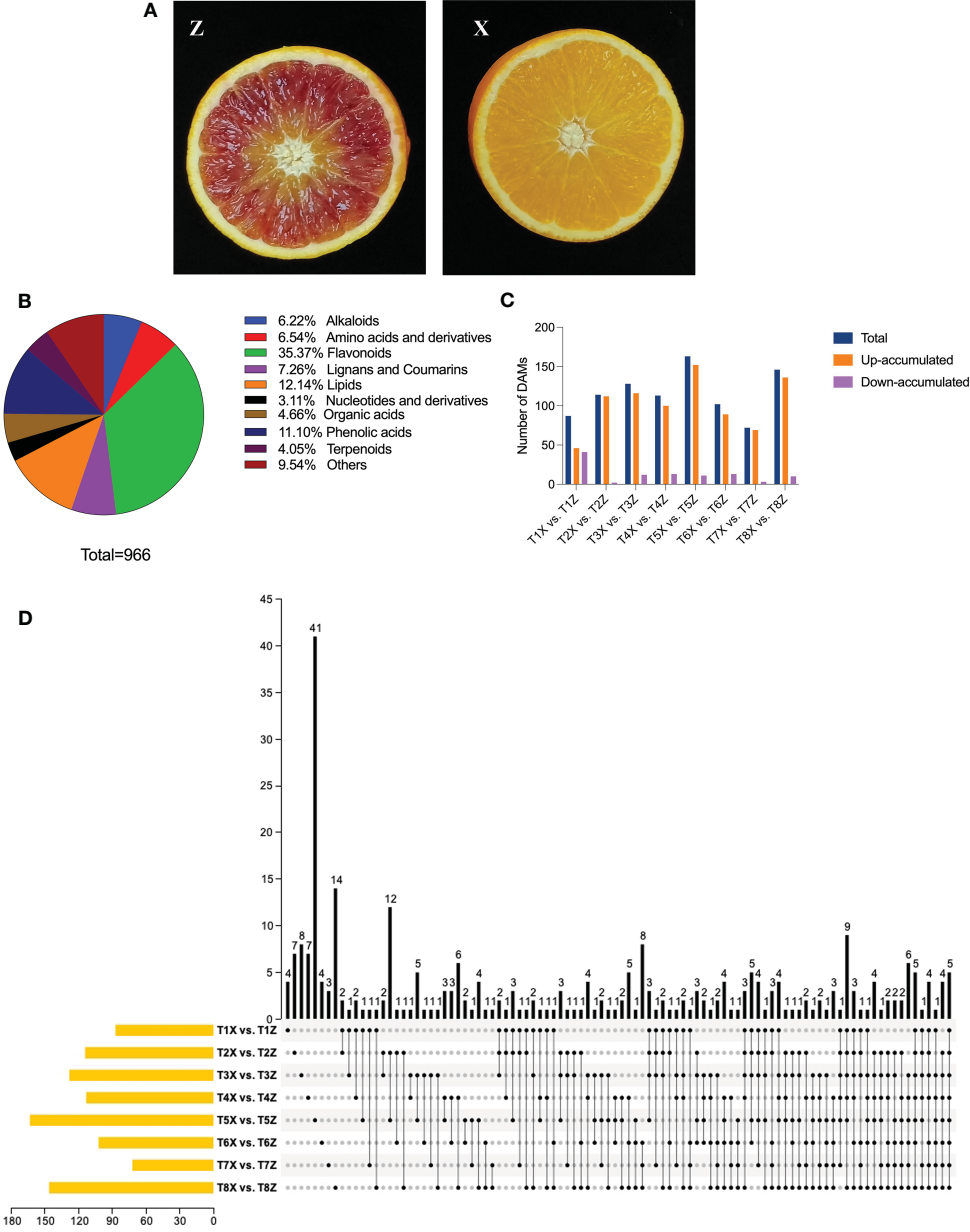
Overview of metabolic profile of lido blood orange on two rootstocks (X and Z) at eight developmental stages (1-8) **(A)** Pictorial description of lido blood orange fruit with rootstocks Z (Trifoliate orange) and X (Ziyang Xiangcheng) **(B)** Identification of metabolites and share of each subclass **(C)** Overview of differentially expressed metabolites between samples at 8 time points (T1-T8) in Lido blood orange with rootstock Z and X **(D)** Upset plot depicting conserved DAMs between different comparisons.

**Figure 2:**
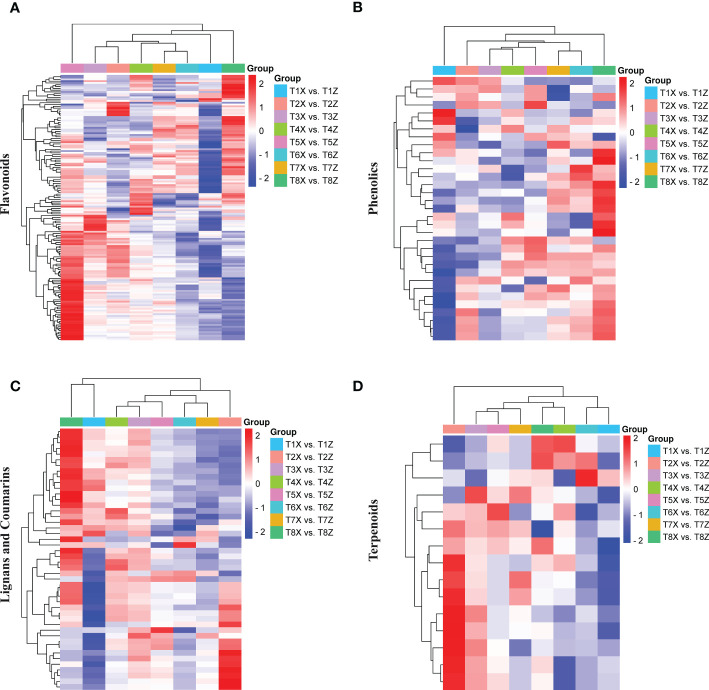
Differential landscape of metabolites in lido blood orange with two rootstocks Z and X **(A)** Metabolic profile of 149 differentially accumulated flavonoids **(B)** Metabolic profile of 34 differentially accumulated phenolic acids **(C)** Metabolic profile of 47 differentially accumulated lignans and coumarins **(D)** Metabolic profile of 17 differentially accumulated terpenoids.

Metabolites, alongside morpho-physiological attributes such as texture, color, size, and shelf life, are crucial determinants of fruit quality. A comprehensive understanding of these factors could provide valuable insights towards improving the nutritional value and marketability of fruits. Therefore, exploring the roles of metabolites and other traits in fruit quality is a crucial step toward promoting human health and enhancing economic benefits in the fruit industry. Metabolome profiling of fruit samples from rootstocks Z and X (**Figure 1A**) at eight different time points were analyzed. The analysis identified 966 metabolites belonging to subclasses of alkaloids, amino acids and derivatives, flavonoids, lignans and coumarins, lipids, nucleotides and derivatives, organic acids, phenolic acids, terpenoids, and others (**Figure 1B** and **Table S3**). Flavonoids were the most abundant metabolites (35.37%), followed by lipids and phenolic compounds. Comparative metabolomics showed significant differences in the accumulation pattern of flavonoids, phenolic acids, lignans and coumarin, and terpenoids in lido blood orange with the two rootstocks (**Figure 2**). A total of 295 differentially accumulated metabolites (DAMs) were identified, with most of them being up-accumulated in Z (**Figure 1C** and **Table S3**).

Five DAMs, namely isomartynoside, prunetin (5,4’-Dihydroxy-7-methoxy isoflavone), deacetylnomilin, isomexoticin glucoside, and deacetylnomilinic acid, belonging to different subclasses showed a conserved up-accumulation pattern at all eight time points (**Figure 1D** and **Table S3**).

Among 341 flavonoids, 149 depicted differential accumulation patterns in at least one comparison (T1-T8) (**Figure 2A**). The significant up-accumulation of most flavonoid compounds in lido blood orange with rootstock Z is highly suggestive that flavonoid accumulation is directly associated with red color formation. Moreover, among the flavonoids, we identified 15 anthocyanins, 1 flavanol, and 1 flavanone (**Figure S4**). The relative quantification of anthocyanins showed an increase with fruit maturity and a higher accumulation in Z compared to X (**Figure S4**).

Among 107 phenolic acids, we only identified 34 differentially accumulated phenolic acids in at least one comparison (**Figure 2B**).

We further characterized subclasses of lignans and coumarins and terpenoids. Almost half of these compounds were differentially accumulated in at least one comparison (**Figures 2C, D**). Overall, the metabolome analysis demonstrated the significant influence of rootstock on the fruit quality in lido blood orange.

### Differential transcriptomic profile of lido blood orange grafted to two rootstocks (Z and X)

**Figure 3:**
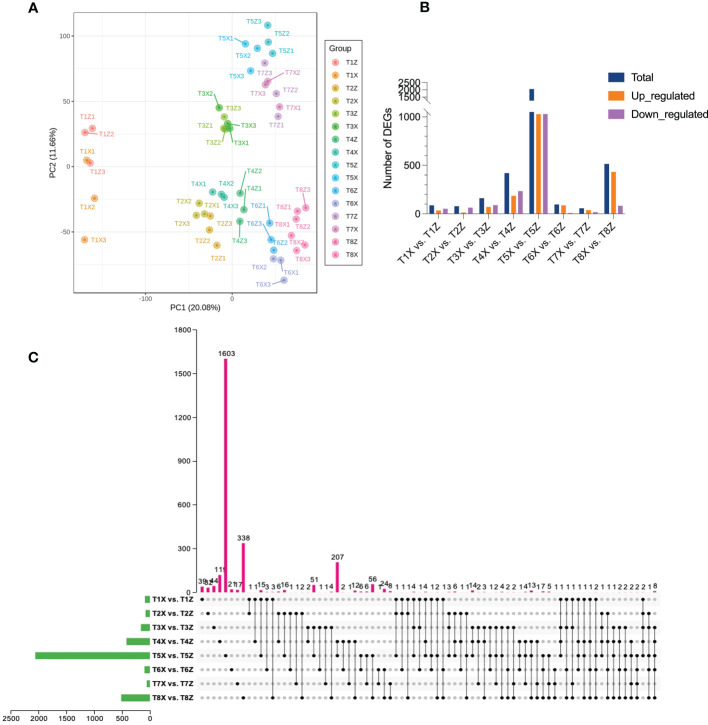
Overview of the transcriptomic profile of lido blood orange on two rootstocks (X and Z) at eight developmental stages (T1-T8) **(A)** Principal component analysis based on FPKM values of fruit samples of lido blood orange with rootstock Z and X at eight time-points **(B)** Overview of differentially expressed genes between samples at different stages **(C)** Upset plot depicting conserved DEGs between different comparisons.

Transcriptome data of X and Z samples from the 8 different time points were analyzed. Expression profiles of 25,701 genes were quantified, generating a total of 457.44 Gb of data. After filtering, 2,799,045,806 (91.79%) clean reads were kept out of 3,049,443,238 raw reads obtained (**Table S4**). The quality check was performed to confer the reliability and reproducibility of the data. Q20 and Q30 were estimated to be over 97% and 92%, respectively (**Table S5**). Moreover, GC contents ranged from 43.27 to 44.68%. All the transcriptome data were subjected to principal component analysis to identify the corresponding variation. PC1 and PC2 covered 20.08% and 11.66% of the variation, respectively, and replicates from each group were clustered, showing the good quality of the transcriptome data (**Figure 3A**).

Pairwise comparisons yielded a total of 4179 differentially expressed genes (DEGs) (**Figures 3B, C**). The expression profiles of all DEGs are presented in **Figure S5** and **Table S6**. Metabolic process and the response to stimulus are the most enriched gene ontology (GO) terms. Next we will investigate changes in specific pathways related to flavonoids, color formation and VC contents.

### Insight into the genes regulating flavonoids, anthocyanin and VC in lido blood orange grafted to X and Z

**Figure 4:**
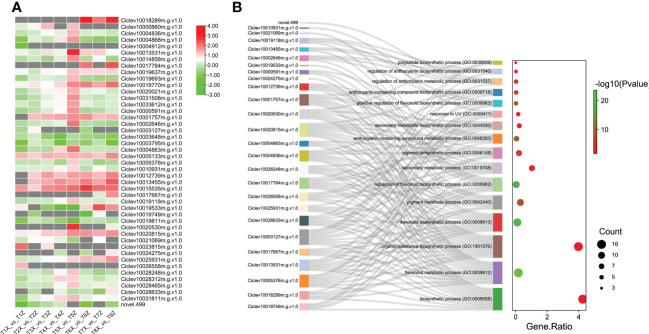
Identification and characterization of DEGs associated with Flavonoid biosynthesis **(A)** Expression profile of 47 DEGs associated with flavonoid biosynthesis and pigmentation at 8 time points (T1-T8) **(B)** GO term enrichment for DEGs associated with flavonoid biosynthesis and pigmentation. The y-axis indicates the GO pathways and associated genes.

Since flavonoids were the major metabolites differentially accumulated between X and Z fruit samples, we further characterized the 45 DEGs involved in flavonoids biosynthesis. Most the DEGs related to flavonoid biosynthesis were upregulated in Z compared to X (**Figure 4**). These results suggest that the rootstock Z increased the expression levels of key flavonoids biosynthesis genes leading to high accumulation of flavonoids in fruits.

**Figure 5:**
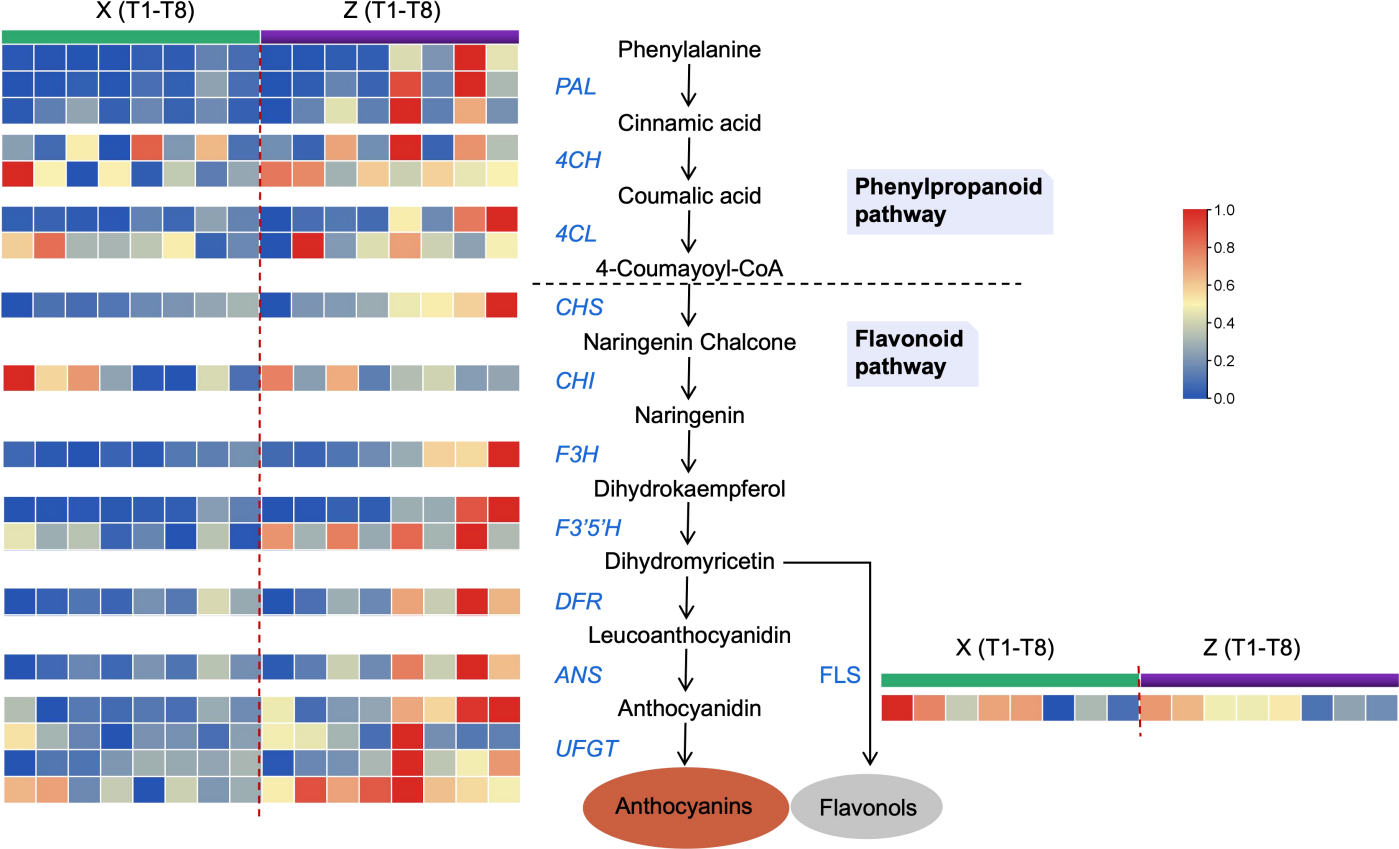
Transcript profiles for genes in the anthocyanin biosynthetic pathway. *PAL*, phenylalanine ammonia-lyase; *4CH*, cinnamic acid 4-hydroxylase; *4CL*, 4-coumarate CoA ligase; *CHS*, chalcone synthase; *CHI*, chalcone isomerase; *F3H*, flavanone 3-hydroxylase; *F3’5’H*, flavonoid 3’,5’ hydroxylase; *DFR*, dihydroflavonol 4-reductase; *UFGT*, UDP-glucose: flavonoid-3-O-glucosyltransferase; *ANS*, anthocyanidin synthase; *FLS*, flavonol synthase. Green and purple represent samples from Z and X, where left to right is the time point from T1 to T8.

Likewise, the DEGs associated with anthocyanin synthesis were further characterized for their potential role in higher accumulation patterns of anthocyanins (blood color formation) in Z. The 17 DEGs showed a differential regulation patterns in Z and X at the 8 time points (**Figure 5** and **Table S7**). *PAL*, *4CH*, *4CL*, *CHS*, *CHI*, *F3H*, *F3’5’H*, *DFR*, *UFGT*, and *ANS* were highly expressed in Z compared to X. Only *FLS* was slightly higher expressed in X compared to Z. It is well known that *FLS* competes with *DFR* to produce flavonols, hence the high FLS expression in X could explain the low anthocyanin content. Overall, the high expression pattern of the major anthocyanin biosynthesis structural genes substantiates the up-accumulation of anthocyanins in Z.

**Figure 6:**
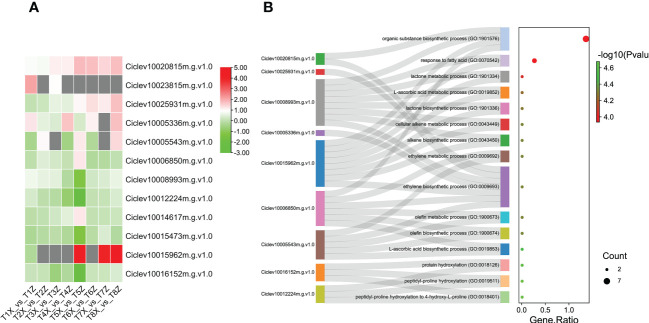
Identification and characterization of DEGs associated with Vitamin C biosynthesis **(A)** Expression profile of 12 DEGs associated with VC biosynthesis at 8 time points (T1-T8) **(B)** GO term enrichment for DEGs associated with vitamin C The y-axis indicates the GO pathways and associated genes.

Furthermore, we screened the 12 DEGs associated with VC biosynthesis based on GO terms. Majority of these DEGs were up-regulated in Z compared to X (**Figure 6** and **Table S6**), which could explain the higher VC content in lido blood orange grafted on rootstock Z as compared to X.

### Gene regulatory mechanisms of anthocyanin accumulation

**Figure 7:**
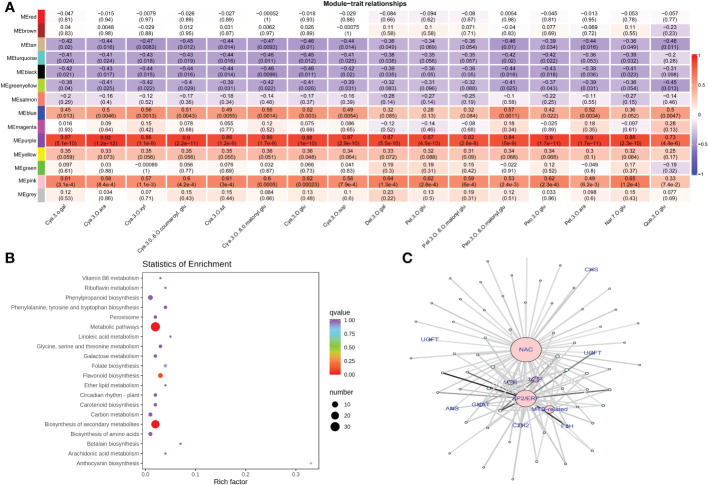
Weighted gene co-expression network analysis for gene mining **(A)** Module/trait correlations and corresponding p values. **(B)** Pathway enrichment analysis of genes in ME-purple module **(C)** The network of the highly connected genes in the ME-purple module. Pink represents transcription factors, and gene names in blue represent the hub genes associated with anthocyanin biosynthesis.

In order to identify regulatory genes involved in anthocyanin accumulation, we performed a weighted gene co-expression network analysis (WGCNA) by constructing a relationship network between DEGs and anthocyanin accumulation. Topology analysis showed that when the threshold β = 13, the scale-free topology fitting index (R2) was close to 90%, indicating that the network was close to a scale-free network (**Figure S6**). Moreover, 13 modules were identified for further screening of hub genes. Module ME-purple showed a significantly higher correlation with anthocyanin accumulation patterns, and therefore we selected the ME-purple module for further assessment (**Figure 7** and **Table S8**). Enrichment analysis of the ME-purple module suggested significant enrichment of pathways, such as metabolic pathways, phenylpropanoid biosynthesis, flavonoid biosynthesis, and anthocyanin biosynthesis (**Figure 7B**). A total of 55 genes were identified in this module with seven transcription factors (*C2H2*, *GANT*, *MYB-related*, *AP2/ERF*, *NAC*, *bZIP*, and *MYB*) and five structural genes associated to the anthocyanin biosynthesis pathway (*CHS*, *F3H*, *UFGT*, and *ANS*). The degree of association of the above-mentioned transcription factors and hub genes has been presented as a network (**Figure 7C**). The network analysis revealed that NAC exhibited the highest connectivity with other genes within the module, suggesting that NAC may play a more prominent role in modulating the anthocyanin content.

### RT-qPCR based verification of expression profile of candidate genes

**Figure 8:**
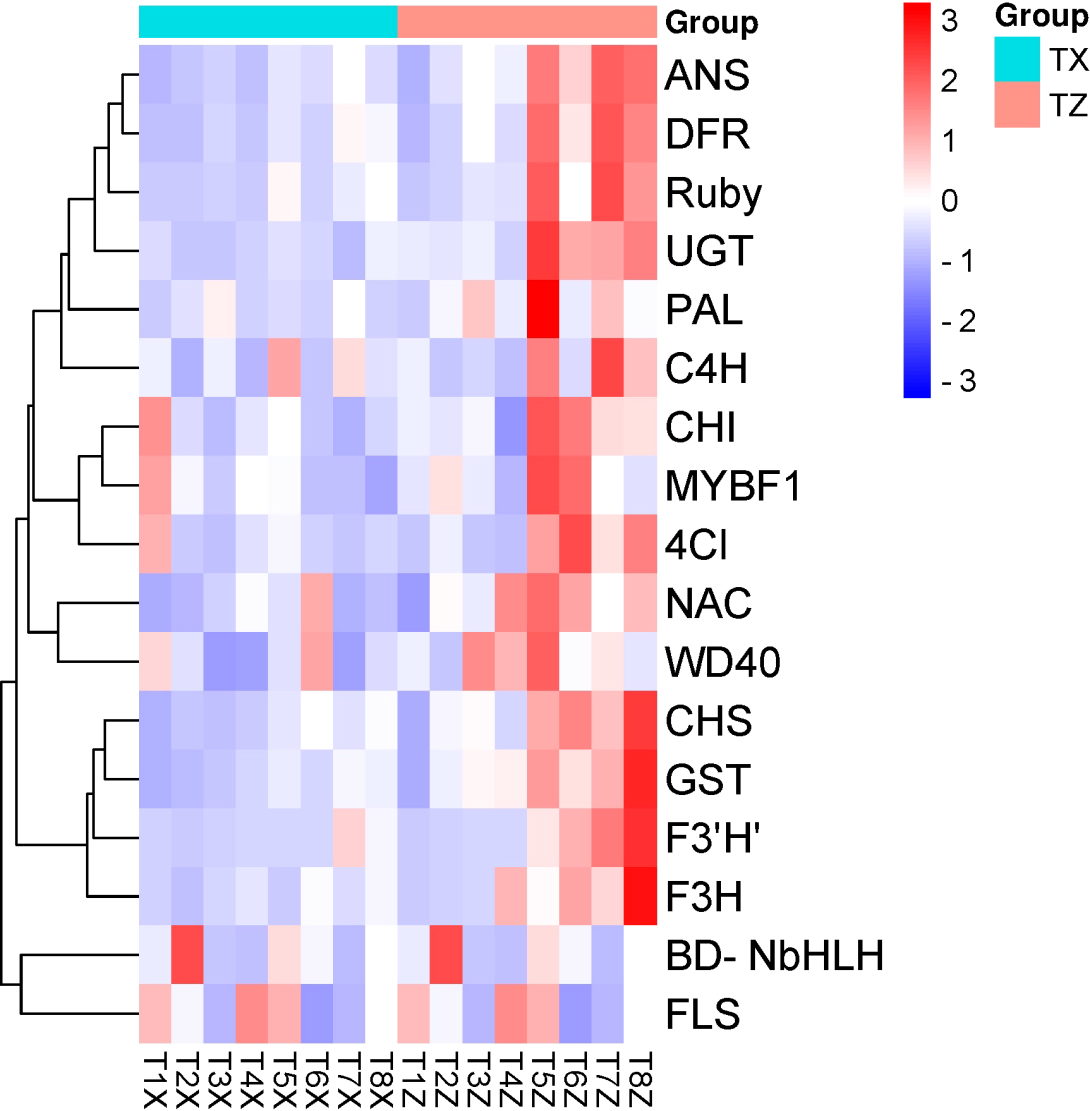
qRT-PCR based expression analysis of selected genes in flavonoid biosynthesis genes at 8 time points (T1-T8) in Lido blood orange with rootstock Z and X. Where *PAL*: Phenylalanine ammonia-lyase, *C4H*: Cinnamate 4-hydroxylase, *4CL*: 4-Coumarate, *CHS*: Chalcone synthase, *CHI*: Chalcone isomerase, *F3H*: Flavanone 3-hydroxylase, *F3’5’H*: Flavonoid 3’,5’-hydroxylase, *DFR*: Dihydroflavonol 4-reductase, *ANS*: Anthocyanidin synthase, *WD40*: WD40 repeat-containing protein, *UFGT*: UDP-glucose: flavonoid 3-O-glucosyltransferase, *GST*: Glutathione S-transferase, *Ruby*: Gene involved in cotton fiber development, *MYBF1*: Myeloblastosis transcription factor 1, *NAC*: NAM, ATAF, and CUC transcription factor family, *FLS*: flavonol synthase, and *BD-NbHLH*: Basic-helix-loop-helix.

A total of 17 candidate genes linked to anthocyanin content in blood orange were used for RT-qPCR experiment. The goal was to validate the expression profile obtained from the RNA-seq. The primers for each candidate gene are listed in **Table S9**. Among the 17 candidate genes, 15 were up-regulated in Z at time points T5-T8 (**Figure 8**). *PAL, C4H, 4CL, CHS, CHI, F3H, F3’5’H, DFR, ANS, WD40, UFGT, GST, Ruby, MYBF1*, and *NAC* were among the 15 up-regulated genes in Z. Moreover, RT-qPCR results correlated well with the RNA-seq data (Pearson correlation R^2 = ^73%), validating the variation observed in the whole transcriptomic datasets.

## Discussion

Blood oranges (*Citrus sinensis* L.) are characterized by a high accumulation of anthocyanins and polyphenolics, conferring their characteristic red color and a valuable source of available antioxidants (Morales et al., 2021). The yield and quality of citrus depend on the genotype, rootstock, and environmental attributes (Cimen and Yeşiloğl, 2016; Martínez-Cuenca et al., 2016; Song et al., 2020; Morales Alfaro et al., 2021). Inherent rootstock differences such as root distribution, water uptake, and hydraulic conductivity are attributed to the final yield and quality of the fruit (Barry et al., 2004). The TSS content in the blood orange juice fell within the 9.47–11.3°Brix range for ‘Z’ and within the 8.65-10.7°Brix range for ‘X’. These values were at par with the previous study concerning ‘Moro’ and ‘Tarocco Rosso’ blood oranges (Morales et al., 2021) and lower than ‘Tarocco Scirè’, ‘Tarocco Rosso’, ‘Tarocco Ippolito’ and ‘Sanguinello’ (12.57 and 15.30°Brix range) (Continella et al., 2018; Cebadera-Miranda et al., 2019). Similar trends were observed for SS and A-conc. Concerning Vitamin C, the results obtained in this study showed a higher concentration of vitamin C (38.5-77 mg/100 ml) in the fruit compared to previous studies ranging from 34.24-66.9 mg/100g (Arena et al., 2001; Rapisarda et al., 2008; Kafkas et al., 2009; Cebadera-Miranda et al., 2019).

To elucidate the metabolome and transcriptome differences pertaining to rootstocks Z (Trifoliate orange) and X (Ziyang Xiangcheng), we systematically evaluated both genotypes at different harvest time points and identified differential regulation of both metabolome and transcriptome. The analysis of differentially accumulated metabolites suggested higher accumulation patterns in ‘Trifoliate orange’ compared with ‘Ziyang Xiangcheng’, emphasizing that lido blood oranges with ‘Trifoliate orange’ rootstock produced superior quality fruit with higher nutrition values. A total of 87% of the differentially accumulated metabolites were up-accumulated in ‘Trifoliate orange’ blood orange. Previously reports also showed significant differences in metabolite accumulation pertaining to different rootstocks. For instance, Miranda et. al., compared Sanguinello and Tarocco blood oranges and identified Tarocco with the highest flavonoid accumulation (Cebadera-Miranda et al., 2019). Another study by Morales et. al., confirmed rootstock-dependent flavonoid accumulation patterns in blood oranges (Morales et al., 2021).

Flavonoids play a crucial role during plant growth and development with various physiological functions such as defense response (Piasecka et al., 2015), post-stress recovery (Havsteen, 2002) and pigmentation (Schiestl and Johnson, 2013). We performed a qualitative and quantitative analysis of secondary metabolites in lido blood orange. Flavonoids were among the most abundant metabolites in this study, and 43% of the flavonoids were identified with differential accumulation patterns. The results are in line with previous reports on metabolite accumulation in blood oranges (Wang et al., 2017). High-performance liquid chromatography has enabled researchers to detect major and minor metabolites of the fruit (Russo et al., 2021). Flavonoids, phenolic acids, and anthocyanins are major characteristic compounds in blood oranges (Lo Piero, 2015). As important subclasses of flavonoids, flavonols, and pro-anthocyanidins are proven to be the main pigment components (Liu et al., 2019), while flavanones are considered major regulators of yellow pigmentations (Peterson et al., 2006; Goulas and Manganaris, 2012). Shi et. al., emphasized that structurally modified anthocyanins and co-pigmented flavonoids are responsible for the purple coloration of tea leaves (Shi et al., 2021). Cyanidin 3-glucoside and cyanidin 3-(6”-malonylglucoside) are considered major anthocyanins in blood oranges, covering 90% of the anthocyanins in blood oranges (Habibi et al., 2022). Various studies have identified other minor anthocyanins in blood oranges, including delphinidin, cyanidin, and peonidin (Lee, 2002). Anthocyanins are also considered a major contributor to the pigmentation of different plant organs (Guo et al., 2009). Reports have emphasized the key role of anthocyanins in tissue color development. For instance, Xue et al. reasoned that red-colored seed-coat formation in peanut is directly associated with anthocyanin accumulation (Xue et al., 2021). Qiu et al. found differential accumulation patterns of anthocyanin in purple fruit and yellow passion fruit (Qiu et al., 2020). Similarly, a study concerning *Primula vulgaris* outlined a gradual increase in anthocyanins as the color deepened from white, yellow, blue, and pink (Li et al., 2020). Similarly, our results emphasized an increased accumulation of anthocyanins in Z (red flesh color) compared with rootstock X (yellow flesh color). Aside from flavonoids, other subclasses of metabolites, including phenolic acids, lignans, coumarins, and terpenoids, were also characterized for their differential cumulation patterns in lido blood orange. Our results signify the differences in metabolite accumulation pertaining to different rootstocks and emphasize that rootstock plays a crucial role in quality enhancement.

Transcriptome provides a genetic basis for a trait under study, and it has been widely used to decipher the genes underlying a specific trait by utilizing contrasting phenotypes (Gusev et al., 2016). Moreover, weighted gene co-expression network analysis can provide significant insights into the co-regulation of trait-specific DEGs (Ding et al., 2021; He et al., 2021; Li et al., 2021). Combined with the metabolome analysis, *chalcone synthase* (*CHS*), *flavanone 3-hydroxylase* (*F3H*), *UDP glucose: flavonoid-3-O-glucosyltransferase* (*UFGT*), and *Anthocyanidin Synthase* (*ANS*) were identified as key structural genes in the flavonoid biosynthesis pathway in Lido blood orange positively regulating the anthocyanin biosynthesis. Wang et. al., observed upregulation of seven co-expressed proteins (*PAL*, *CHS*, *F3’H*, *F3’5’H*, *DFR*, *ANS*, and *UFGT*) and their corresponding mRNAs (Wang et al., 2017). Interestingly, there were strong positive correlations between the transcription levels of these genes and the protein expression levels. Previously reports are highly suggestive of the active involvement of *CHS* and *UFGT* in regulating anthocyanins in different plant tissues (Hu et al., 2022; Mao et al., 2022; Meng et al., 2022; Muhammad et al., 2022; Ohta et al., 2022). Castellarin et. al., while studying red grapes, reported that the expression of *Flavonoid 3’,5’-hydroxylase* controls the production of red cyanidin-based anthocyanins accumulated as red pigments (Castellarin et al., 2006). Several other reports also suggested the significant role of *Flavonoid 3’,5’-hydroxylase* in pigmentation (De Vetten et al., 1999; Boase et al., 2010; Ishiguro et al., 2012; Vikhorev et al., 2019). Moreover, several transcription factors (TF), including *C2H2*, *GANT*, *MYB-related*, *AP2/ERF*, *NAC*, *bZIP*, and *MYB*, were also identified as a regulators of hub genes in this study. Our results suggested that the NAC TF might play a key role in anthocyanin synthesis. Although several studies have demonstrated that the *NAC* family is involved in the regulation of various biological processes, from plant development to response to stress, a few members have been identified as regulators of anthocyanin biosynthesis (Morishita et al., 2009; Zhou et al., 2015; Mahmood et al., 2016; Jiang et al., 2019; Zhang et al., 2020; Martín-Pizarro et al., 2021). For instance, in peach (*Prunus persica*), a *BLOOD* (BL) gene encoding a *NAC* TF played a key role in determining the blood-flesh trait by activating *PpMYB10.1* in the process of peach fruit ripening (Zhou et al., 2015). Similarly, Zhang et al. identified a *NAC* TF *MdNAC42* in red-fleshed apples, which could interact with *MdMYB10*, an important positive regulator of anthocyanin biosynthesis, to promote anthocyanin accumulation during apple fruit ripening (Zhang et al., 2020). Zou et al. demonstrated that silencing *LcNAC002* led to a significant reduction in the expression levels of *LcSGR* and *LcMYB1*, inhibiting chlorophyll degradation and anthocyanin accumulation in *Litchi chinensis* (Zou et al., 2023). Another study by Morishita et. al., demonstrated that *LcNAC13* physically interacts with *LcR1MYB1* to coregulate anthocyanin biosynthesis-related genes during litchi fruit ripening (Jiang et al., 2019). In strawberry, *FaNAC035* could induce anthocyanin accumulation during fruit ripening by promoting ABA accumulation (Martín-Pizarro et al., 2021). Liu et al. reported that *MdNAC1* expression is significantly upregulated in response to abscisic acid (ABA) due to an ABRE cis-acting element in its promoter region. Furthermore, the accumulation of anthocyanins in apple co-transformed with *MdNAC1* and *MdbZIP23* was observed to be enhanced in the presence of ABA (Liu et al., 2023). These findings suggest that *MdNAC1* may be involved in the ABA-mediated regulation of anthocyanin biosynthesis in apple. Since the molecular mechanism of NAC TFs in citrus is poorly understood, it is important to investigate whether they can form protein complexes with other transcription factors like MYB, WD40, or bHLH to regulate anthocyanin synthesis.

Citrate accumulation is the main reason for the acidity of citrus fruits. However, the decrease of acidity during the ripening stages is associated with the catabolism of citrate (Cercós et al., 2006). *NAD(P)* is a key gene family involved in citric acid catabolism, and its upregulation results in reduced citrate accumulation (Guo et al., 2016). The upregulation of NAD(P)-dependent IDH results in increased conversion of isocitrate to α-ketoglutarate, which leads to reduced citrate accumulation in the TCA cycle. This is because isocitrate is a precursor of citrate, and its rapid conversion to α-ketoglutarate reduces the amount of citrate produced in the TCA cycle. Therefore, the upregulation of NAD(P)-dependent IDH can modulate citric acid catabolism and ultimately affect citrate accumulation in the cell. Our results suggested up-regulation of both *ATP citrate synthase* and *NAD(P)-bd_dom domain-containing protein* at harvest time-point 8 (T8). Together with the pattern of acid concentration in lido blood orange with different rootstocks, regulation of these genes is highly suggestive of playing a pivotal role in higher acid accumulation in lido blood orange with rootstock Z compared to X.

## Conclusion

Together, physiological, metabolic, and transcriptomic characterization revealed that lido blood orange with ‘Trifoliate orange’ rootstock is superior in fruit quality compared with ‘Ziyang Xiangcheng’. Our results also provided key regulators for flavonoids, anthocyanins, and acidity, which can be a valuable source for further utilization in improving blood orange varieties.

## Data availability statement

The raw RNA-seq datasets used for this study can be found in the NCBI SRA under the project number: PRJNA898909 (https://www.ncbi.nlm.nih.gov/bioproject/?term=PRJNA898909).

## Author contributions

Conceptualization, LY, LH, YC, and MW; Formal analysis, YC, Shuang Li, and LG; Funding acquisition, LH; Investigation, HY and HH; Methodology, WW and MW; Resources, YC; Software, HH; Supervision, LY; Validation, YC and LG; Visualization, HY; Writing – original draft, LH and LY; Writing – review and editing, LH and LY. All authors contributed to the article and approved the submitted version.

## Supplementary material

Supplementary Figure 1 Mean comparisons of physiological parameters of Lido blood orange on four rootstocks at eight time points **(A)** Total soluble solids (TSS), **(B)** Acid concentration, **(C)** Vitamin C, and **(D)** soluble solids to acidity ratio (TSS/Acid concentration).

Supplementary Figure 2 Mean comparisons of color parameters of Lido blood orange on four rootstocks at eight time points **(A)** L (brightness), **(B)** a (red/green value), and **(C)** b (Blue/yellow value).

Supplementary Figure 3 Pictorial description of lido blood orange fruit with rootstocks **(A)** Z (Trifoliate orange) **(B)** ZC (Citrange), **(C)** H (*C. reticulata* Blanco), and **(D)** X (Ziyang Xiangcheng).

Supplementary Figure 4 Anthocyanin accumulation pattern in lido blood orange with Z and X rootstocks at eight time points.

Supplementary Figure 5 Expression profile of identified DEGs in multiple comparisons of lido blood orange with Z and X rootstocks at eight time points.

Supplementary Figure 6 Weighted gene co-expression network analysis **(A)** cluster dendrogram **(B)** Eigengene adjacency heatmap **(C)** Topology analysis of appropriate weighting coefficient **(D)** mean connectivity.
