# Supplementary figures and images for: Metabolomic and transcriptomic analyses reveal the effects of grafting on blood orange quality
Source: Front Plant Sci. 2023 Jun 1;14:1169220. doi: 10.3389/fpls.2023.1169220 (PMC10286243; doi:10.3389/fpls.2023.1169220)

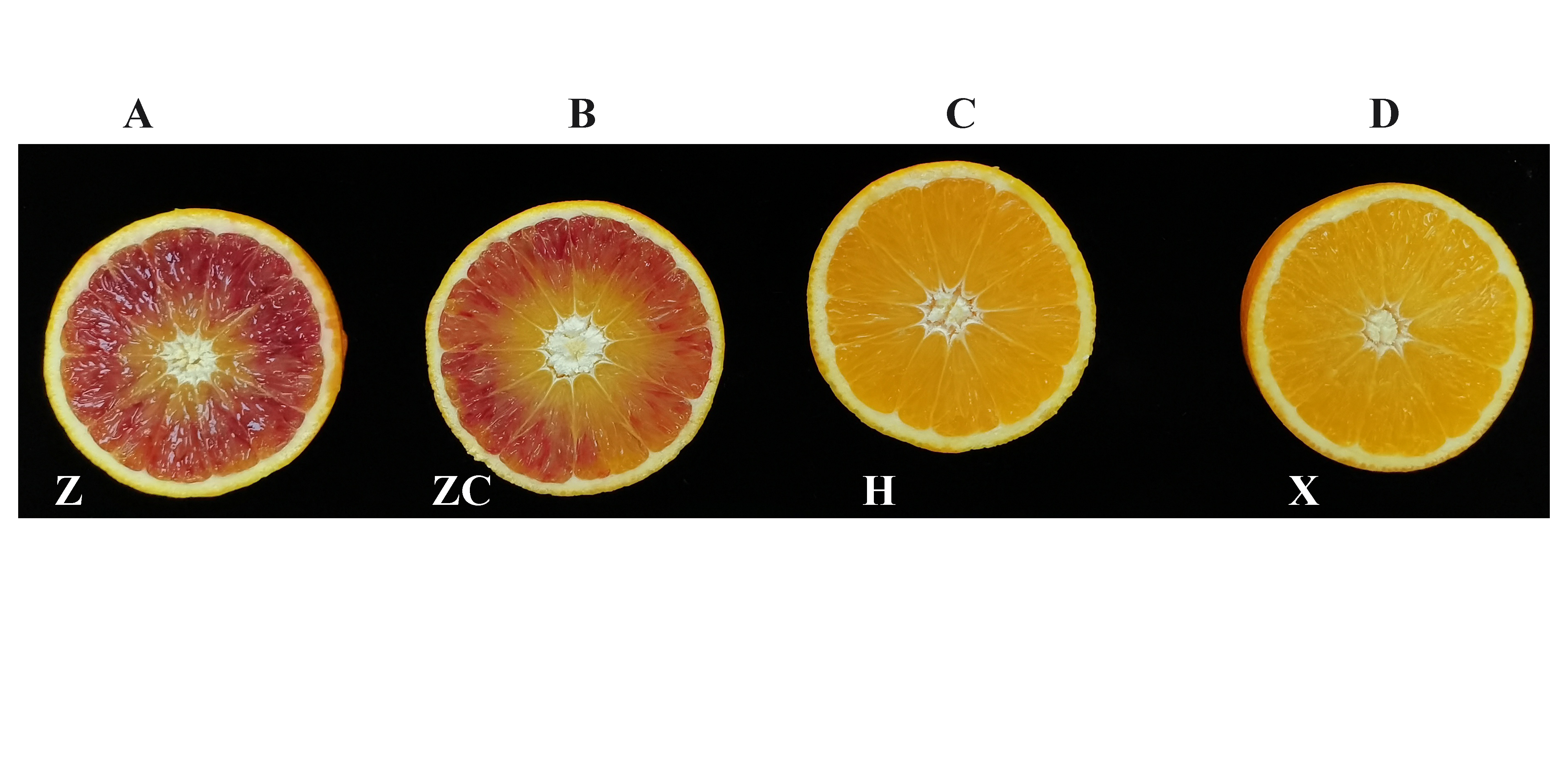

Supplement: Supplementary Figure 3 — Pictorial description of lido blood orange fruit with rootstocks (A) Z (Trifoliate orange) (B) ZC (Citrange), (C) H (C. reticulata Blanco), and (D) X (Ziyang Xiangcheng). [file Image_3.jpeg]

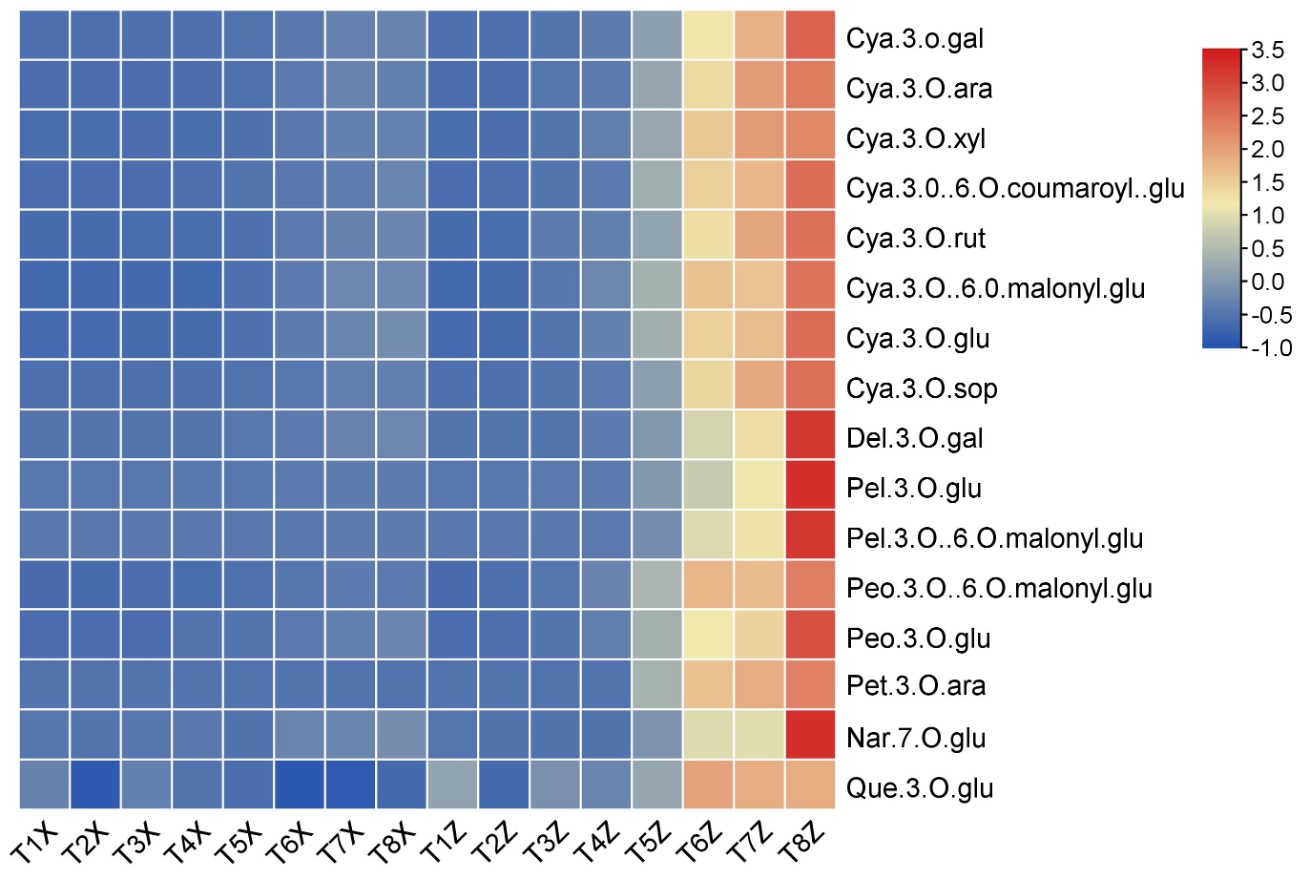

Supplement: Supplementary Figure 4 — Anthocyanin accumulation pattern in lido blood orange with Z and X rootstocks at eight time points. [file Image_4.jpeg]

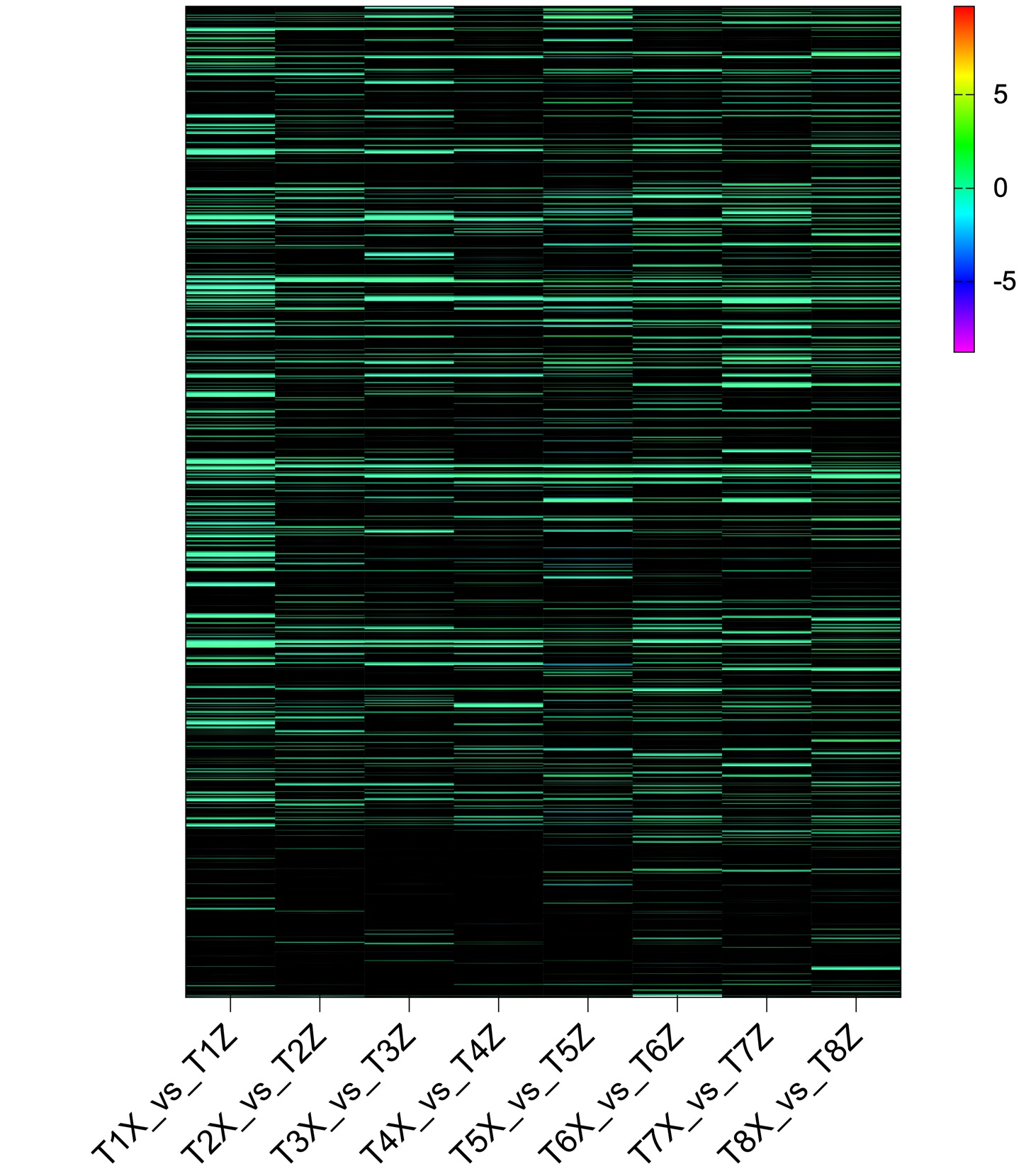

Supplement: Supplementary Figure 5 — Expression profile of identified DEGs in multiple comparisons of lido blood orange with Z and X rootstocks at eight time points. [file Image_5.jpeg]

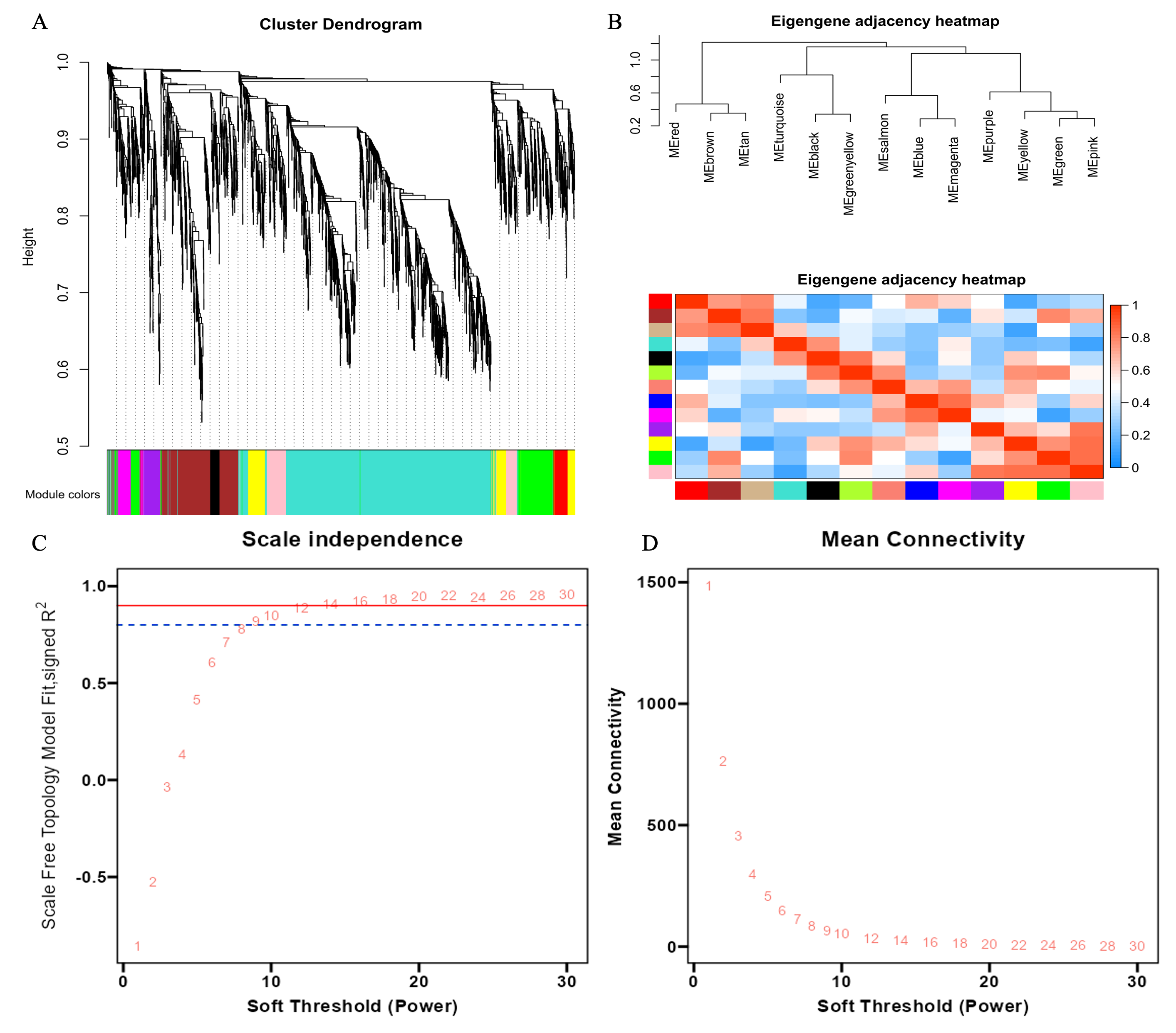

Supplement: Supplementary Figure 6 — Weighted gene co-expression network analysis (A) cluster dendrogram (B) Eigengene adjacency heatmap (C) Topology analysis of appropriate weighting coefficient (D) mean connectivity. [file Image_6.png]
